# Racism, Colorblindness, and Police Culture in Canada

**DOI:** 10.1177/21533687251377225

**Published:** 2025-09-16

**Authors:** Manzah-Kyentoh Yankey

**Affiliations:** Department of Sociology, 3158 University of Alberta, Edmonton, AB, Canada

**Keywords:** police culture, policing, racism, colorblind racism

## Abstract

Despite the amount of research on police culture, little is known about how police culture reinforces systemic racism in Canadian policing. In race and policing scholarship, less is known about how women police officers perpetuate systemic racism in policing. Based on 16 interviews with women police officers from a police organization in Alberta, this study examines how police culture reinforces systemic racism in Canadian policing. Using colorblind racism and intersectionality, the findings demonstrate that officers regularly say racist jokes to normalize racialized police violence. Officers emphasize warrior police culture and suspiciousness to physically assault and racially profile Indigenous people, including those living in encampments. Officers associate Blackness with criminality by reinforcing culturally racist stereotypes about Black Canadians, such as having criminal lifestyles. Furthermore, officers hold racist and xenophobic perceptions about refugees and when refugees call the police for help, officers culturally frame them as criminals and blame all refugees for an individual’s criminal offense. White women officers were more likely than Black women and Biracial (Indigenous/White) women officers to reinforce the colorblind racist myth that racialized police violence is only an American problem, and that policing is a race-neutral practice.

## Introduction

Systemic racism in policing is intertwined with the historical and contemporary legacy of colonialism, slavery, racial capitalism, and White supremacy (Hinton & Cook, 2021; Samuels-Wortley, 2024; Stelkia, 2020; Steinmetz et al., 2017). Racial justice movements such as the Black Lives Matter movement in the United States, Canada, and globally have centered on the impact of systemic racism in policing and police violence against people of color, generating widespread calls for police reform, police transformation, and police abolition (Cobbina-Dungy & Jones-Brown, 2023; Cunneen, 2023). However, despite calls for systemic change, criminal legal institutions tend to adapt colorblind strategies for addressing systemic racism in the Canadian and American criminal legal system (Alexander, 2020; Dunlea, 2022; Samuels-Wortley, 2021; Van Cleve, 2016). Policing institutions tend to abide to the “bad apples” paradigm, claiming the problem of racism in policing is the individual actions of a few police officers and not the system (Jones et al., 2023, p. 1). Scholars argue that adapting a colorblind approach to addressing racism worsens the overrepresentation of Black, Indigenous, Latine, and other people of color in the Canadian and American criminal legal system (Alexander, 2020; Samuels-Wortley, 2021; Van Cleve, 2016).

Research based in the United States, Canada, and the United Kingdom show that Black, Indigenous, and people of color disproportionately experience police use of force, stop and search, police sexual violence, and police arrests (Alberton et al., 2019; Bundy, 2019; Campagna & Zaykowski, 2024; Cobbina-Dungy & Bailey, 2024; Comack, 2012; David & Mitchell, 2021; Duhaney, 2022a, 2022b; Epp et al., 2014; Flores & Alfaro, 2023; Laming, 2023; Long, 2018; Maynard, 2017; Owusu-Bempah & Gabbidon, 2021; Palmater, 2016; Razack, 2015; Rowe, 2004; Rudin, 2006; Samuels-Wortley, 2021, 2022a; Wortley & Owusu-Bempah, 2011, 2022). Police culture plays a significant role in perpetuating systemic racism in policing (Bell, 2018; Chan, 1997; Foster, 2008; Pérez & Ward, 2019; Roscigno & Preito-Hodge, 2021; Smith & Grey, 1985). However, fewer studies capture how police culture reinforces systemic racism in Canadian policing (Satzewich & Shaffir, 2009; Tator & Henry, 2006). In race and policing scholarship, White women and Women of Color officers perceptions of systemic racism in policing remain largely unexamined, as the focus has primarily been on White men (Carlson, 2020; Glover, 2007; Whitehead, 2015), the dominant group in police organizations because of White supremacist patriarchy (Bikos, 2023).

This article examines how police culture reinforces systemic racism in Canadian policing. Using colorblind racism and intersectionality, I draw on 16 interviews with women police officers from a police organization in Alberta to address the following research question: *How does police culture reinforce systemic racism in Canadian policing?* In the following sections, I review the literature on police culture and theoretical frameworks colorblind racism and intersectionality. I then provide details about my methodology and findings. I conclude the article by discussing the implications of these findings for understanding how police culture reinforces systemic racism in Canadian policing.

## Police Culture

Since the 1960's, decades of ethnographic work on police culture in the United States, United Kingdom, New Zealand, and Australia have examined how officers interpret the occupational norms and values within the bureaucratic structure of police organizations (Chan, 1997; Loftus, 2010; Sierra-Arevalo, 2019, 2021). Police culture research in the 1960s and 1970s illustrated that police officers often expressed the need for solidarity and discipline with officer colleagues in police-community encounters (Banton, 1964; Bittner, 1970; Manning, 1977; Skolnick, 1966; Van Maanen, 1974, 1975, 1978; Westley, 1970). However, the authority officers enforced on communities made them a socially isolated group, resulting in an “us versus them” mentality (Banton, 1964; Skolnick, 1966). In response to this potential danger, officers develop a “working personality,” which socializes officers to be skeptical and suspicious toward the communities they police and arrest individuals who break the law (Skolnick, 1966, p. 42).

Police culture is a set of cultural values and norms that influence how officers perform their assigned tasks and duties (Chan, 1996, 1997; Crank, 2014; Ingram et al., 2013; Loftus, 2008; Paoline, 2003, 2004; Paoline & Gau, 2018; Reiner, 2010; Waddington, 1999). Police culture emphasizes strong solidarity with officer colleagues, misogyny, racism, suspiciousness, and conservatism (Reiner, 2010). Police culture includes a “warrior-like” mindset (Sierra-Arevalo, 2019, 2021; Simon, 2023; Stoughton, 2014) that glorifies violence, aggressive masculinity, abuses authority and is extremely resistant to accepting community criticism and police reform (Fielding, 1994; Herbert, 2001; Manning, 2005; Paoline et al., 2006; Westley, 1970). The culture of authoritarianism in policing creates a “blue wall of silence” (Bittner, 1970; Westley, 1970) that damages police accountability and helps officers avoid organizational punishment for police misconduct, ranging from excessive use of force, wrongful arrest to murder (Marenin, 2016; Sierra-Arevalo, 2021; Skolnick, 1966). Officers who break the culture of silence by reporting an officer for misconduct often face retaliation from the police organization (Nhan, 2014).

Police culture also perpetuates systemic racism in policing (Bell, 2018; Chan, 1997; Foster, 2008; Pérez & Ward, 2019; Roscigno & Preito-Hodge, 2021; Satzewich & Shaffir, 2009; Smith & Grey, 1985; Wortley et al., 2020). Police officers often depict people of color as dangerous criminals who are extremely violent and irrational (Pérez & Ward, 2019; Wortley et al., 2020). Bell (2018) noted the importance of Skolnick's (1966) concept of the symbolic assailant to understanding the ongoing police violence of Black men and women in the United States. The “symbolic assailant” is a person whose “gesture, language and attire the policeman has come to recognize as preclude to violence” (Skolnick, 1966, p. 45). Considering the several murders of Black people by American police departments, and racialized policing practices (e.g., Broken Windows, Stop and Frisk, No-Knock Police Raids, Gang Suppression, Police Surveillance, Excessive Use of Force), Bell (2018) argues that Blackness itself is the symbolic assailant, where Black men, regardless of age, ethnicity, gender, class, and neighborhood context, are always perceived as dangerous, “suspicious and up to no good” (Bell, 2018, p. 534). Black women experience police violence, especially in situations where they have a mental health disability, “advanced age, and when they are in the presence of young children,” resulting in a serious injury, or even death (Bell, 2018, p. 548). The concept of the Black symbolic assailant, according to Bell (2018), starts as early as childhood, as Black youth are “criminalized and placed into the ‘school-to-prison pipeline’” (Bell, 2018, p. 542).

Little is known about how police culture reinforces systemic racism in Canadian policing (Satzewich & Shaffir, 2009; Tator & Henry, 2006). Based on 18 semistructured interviews with Canadian police officers from various gender and racial backgrounds, Satzewich and Shaffir (2009) find that police officers repeatedly deny racism in Canadian policing, arguing that racial profiling is an important part of police work. When police culture is applied to understand racism in policing, racial profiling “occurs even in the absence of officers who may be inclined to prejudice or discrimination against members of visible minorities” (Satzewich & Shaffir, 2009, p. 201). Most police officers in Satzewich and Shaffir (2009) study denied racism in Canadian policing by referring to changes to police organizations equity, diversity, and inclusion policies, claiming that racism is no longer apart of Canada (Satzewich & Shaffir, 2009). Officers often pointed to Canadian multiculturalism policies and practices to deny racism, arguing that younger police officers grew up in racially diverse communities and “have gone to school with other students of diverse backgrounds” (Satzewich & Shaffir, 2009, p. 216). Officers denied allegations of racism by suggesting that people of color often play the “race-card” to prevent officers from assessing their criminal behavior (Satzewich & Shaffir, 2009, p. 217).

## Colorblind Racism and Intersectionality

Colorblind racism is a racial ideology that denies and minimizes racism by articulating race-neutral beliefs about, for example, individualism, culture, neoliberalism, economic wealth and politics to justify racial inequality (Burke, 2019). Bonilla-Silva (2022) identifies four central frames of colorblind racism: “abstract liberalism, naturalization, cultural racism, and minimization of racism” (Bonilla-Silva, 2022, p. 80). Abstract liberalism uses ideologies associated with individualism to deny racism (Bonilla-Silva, 2022). Naturalization is a frame popular in White racial discourse that portrays racism as a natural coincidence unrelated to race (Bonilla-Silva, 2022). Cultural racism relies on culturally racist stereotypes such as Black children are criminals to explain racial inequality (Bonilla-Silva, 2022). Minimization of racism suggests that racism is no longer an issue affecting the living conditions of racialized communities (Bonilla-Silva, 2022). Colorblind racism fundamentally structures white supremacy in police organizations (Delgado, 2018). In policing, officers use colorblind ideologies to deny or minimize the reality of systemic racism in policing (Delgado, 2018; Gordon, 2024; Jones et al., 2023; Kim, 2024; Welsh et al., 2021). Police officers often claim that policing practices, such as police shootings, racial profiling, stop and search is colorblind, while incorporating racist narratives about people of color (Gordon, 2024; Welsh et al., 2021). Although Bonilla-Silva's (2022) central frames of colorblind racism is written in the American context, the theory is useful for understanding colorblind racism in the Canadian context, including how Canadian police officers underestimate or deny race as a central part of their work.

Intersectionality provides a framework for understanding how the intersection of racism, sexism, classism, and other oppressive forms of power reproduces social inequality (Cho et al., 2013; Collins, 2019; Collins & Blige, 2020; Crenshaw, 1989, 1991). An intersectional framework on race, gender, sexuality, colorblind racism and other interlocking power structures (Carbado, 2019; Ferber, 2007; Frankenberg, 1993) constructs police officers perceptions of systemic racism in policing (Carlson, 2020; Glover, 2007; Whitehead, 2015). Carlson's (2020) research on race, masculinity, gun violence, and policing in the United States found that White male police chiefs minimize the victimization of gun violence affecting Black and Brown boys and men by constructing the victims and perpetrators as gangsters and drug-dealers. In contrast, White male police chiefs perceive gun violence against White people as an active-shooting related incident (Carlson, 2020). White male police chiefs associate the masculine “warrior” police culture with aggressive policing towards Black and Brown boys and men and the “guardian” culture with protecting White victims (Carlson, 2020, p. 413).

Glover (2007) found patterns of colorblind racism in the way White male officers frame racial profiling. For example, some White male officers would use the “White boy in a no white boy zone” narrative: a White person walking in a predominantly racialized neighborhood to downplay racial profiling against people of color in the United States (Glover, 2007, p. 243). White male officers in Whitehead's (2015) ethnographic study dismissed Black Americans concerns about anti-Black racism in policing and the overpolicing of their neighborhoods during routine traffic stops. Instead of acknowledging systemic racism in policing, White male officers claim to have racial anxieties because they fear being called a racist officer, completely ignoring racial disparities in policing practices (Whitehead, 2015). In this study, I use colorblind racism and intersectionality to explore women officers perceptions of how police culture reinforces systemic racism in Canadian policing.

## Methodology

Between February and April 2021, I conducted 16 semistructured interviews with women police officers from a police organization in Alberta. I gained access to the police organization through the police organization's research unit. The police research unit forwarded my recruitment letter to women police officers, including 28 Women of Color and 4 LGBTQIAA2S+ officers, inviting them to participate in the study. About half of the 16 interviews were conducted on the phone and the other half were conducted on Zoom. Of the 16 participants, 12 identified as White, three identified as Black and one identified as Biracial (Indigenous/White). Of the three Black women officers involved in the study, two are Biracial (White/Black), but racially identify as Black. Four White women identified as openly gay (LGBTQIAA2S+). The average number of years in policing for women officers who participated in the study was 14 years, with a range of five to 24 years of policing experience Table 1.

**Table 1.** Interview Participants (*n* = 16).

Race/gender	*N*	Percentage
White women	12	75
Black women	3	18
Biracial women (Indigenous/White)	1	6

I employed a “semistructured” interview style, so that participants can respond directly to questions but still feel comfortable bringing up other points for discussion (Warren, 2001). Interviews lasted, on average, between 45 and 60 min. My interview prompts included questions about police culture, racism in policing, and police–citizen encounters, paying particular attention to how the racialized aspects of police culture are characterized and divided among officers. With the consent of my participants, I audio-recorded and transcribed verbatim after each interview. I anonymized the participants names and use pseudonyms throughout the analysis.

Throughout the data collection stage, I continuously analyzed interviews by frequently re-reading interview transcripts and identifying relevant themes and categories discussed by women police officers. I independently coded and analyzed each interview using NVivo 12 software. I used a multistep coding procedure, as outlined by Saldana (2016). First, I conducted an intercoder reliability check by dividing the interview transcripts into different sections, carefully analyzing and identifying various themes and categories (Charmaz, 2014; Strauss & Corbin, 1998). Through “open coding” (Saldana, 2016, pp. 115–119), I identified thirteen broad categories, including: “Racism, Police Culture, and Police-Citizen Encounters.” To ensure coding quality and reliability, I re-read the transcripts at least twice and created more categories and subcategories. For example, under the Racism code, I created the following subcategories: “Systemic Racism, Overt Racism, Racist Jokes, Anti-Indigenous Racism, Anti-Black Racism, Racism/Xenophobia, and Colorblind racism.” I then used an axial coding approach by identifying relationships among the categories and subcategories I documented during the earlier and later stages of qualitative data analysis (Saldana, 2016).

I note that my researcher positionality as a cis-gendered Black woman graduate student who has never worked for any police organization shaped the scope of this research. My positionality negatively affected my experiences with getting access to the police organization. After receiving research ethics approval from the university in July 2020, I reached out to the police to start recruiting participants. Shortly after attempting to recruit participants, the organization started gatekeeping my research project. After the high-profile police murders of George Floyd and Breonna Taylor in the United States and Regis Korchinkski-Paquet in Canada, the police organizations research unit became suspicious about my Blackness and constantly postponed my request to recruit women officers. Given that police culture is deeply rooted in patriarchy, racism, and queerphobia (Bikos, 2023; Miles-Johnson, 2020; Preito-Hodge, 2020), getting access to police organizations can be challenging for external academic researchers who don’t have strong partnerships with the police (Cheng, 2025), especially researchers who are not White middle-class men and women (Samuels-Wortley, 2022b; Vera Sanchez & Portillos, 2021). After numerous attempts to determine the status of my request, the police organization granted me access in mid-December 2020, and I began conducting interviews in February 2021. The challenges I experienced with getting access to the police department is an illustration of how Canadian police services are racialized organizations (Ray, 2019) who use state institutional power and whiteness to control which researchers can interview police officers.

My positionality shaped my interactions with participants during interviews. Although most women officers were comfortable speaking to me about police culture and racism, some participants, especially White women, were suspicious about my intention for conducting the study. For example, one White woman officer during the phone interview accused me of viewing the police organization as a racist institution and unsafe place for women to work because I’m Black “*I’m afraid that like, what you'll read is that this place is completely racist and bad and it's bad for women, right? Because that's not necessarily true, either … I don't want to study like this to blow it out of the water. I don't know what other people are telling you.*” At the end of the interview, the officer started yelling at me and admitted that she intentionally hid information about the police because she doesn’t want to throw the organization under the bus “*I wasn't very specific … But I feel like I also don't want to throw the organization under the bus too, because they're still trying to change!!!.*” The White woman officer interpreted my race and gender as a threat to the institution. Some participants asked me racially stereotypical questions about my perceptions and experiences of the police. One White woman officer asked if I had any previous interactions with the police. I briefly said I had a hostile encounter with a White woman officer in the past. After I answered her question, she immediately denied racism and said, “*that's an individual issue, not a police issue.*” Not only did my positionality influence my data collection, but also my data analysis. My positionality influenced how I coded the interview data, feature interviewees narratives to demonstrate my claims and structure my arguments throughout the analysis.

## Findings

Below, I discuss four themes that emerged from my analysis of interviews: Racist Jokes, Anti-Indigenous Racism, Anti-Black Racism, Xenophobia and Racism. I begin by explaining how officers say racist jokes to justify racialized police violence and the way White women officers use the minimization of racism frame to underestimate the severity of racial humor in police culture. Second, I describe Black women and White women officers’ discussions about how warrior police culture and suspiciousness shapes anti-Indigenous racism in Canadian policing. Third, I discuss women officers’ perceptions about anti-Black racism in policing and how White women use cultural racism to deny anti-Blackness in Canadian policing. Lastly, I discuss about how police culture fuels xenophobia and racism against refugees.

### Racist Jokes

Women officers reported that police officers regularly say racist jokes at work. Officer Amelia (Black), said some officers say racist and Islamophobic comments about Muslims by jokingly calling each other “*Jihadists*,” firing gunshots: *“There's some people that would just jokingly greet themselves by screaming, ‘Jihad’ and making gunshots and I was like ‘what the hell.’ So inappropriate. First of all, there are some certain things that are said that are very inappropriate.*”

Officer Sophia (Black) noted that officers regularly say racist jokes that are at times directed toward Indigenous people: “*sometimes there's jokes, there's always going to be jokes that some members make that are racially, you know, kind of sprinkled with a bit of racism, not necessarily directed to me, but maybe to Indigenous people.*” Officer Caitlin (White, LGBTQIAA2S+) said officers often say racist jokes in the typing room. “*In our typing room, there's all sorts of racial jokes and stuff that are said all the time. And you know, people there not aware of who's around them. And, you know, just making jokes about very racially stereotypical inappropriate stuff.*”

The examples Black women and White women LGBTQIAA2S+ officers mention illustrates how officers use racist jokes such as calling each other “*Jihadists*” and firing gunshots to normalize racialized police violence, which helps maintain white supremacy in police culture (Pérez & Ward, 2019). Criminologists often claim that police culture builds solidarity among police officers (Waddington, 1999). However, the findings in this study show that police culture normalizes racism in policing because officers circulate racist jokes throughout the police organization (Cashmore, 2001; Pérez, 2022; Reiner, 2010).

Some White women officers reveal they’ve seen officers say racist jokes at work and yet argue that systemic racism in Canadian policing is less harmful than American policing. White women officers in this study use the minimization of racism colorblind frame (Bonilla-Silva, 2022) to downplay racism in Canada. Officer Genevieve (White) spoke about the police killings of Black Americans in the United States. She claims that compared to the United States, Canadian police officers treat racialized communities better than American police officers because of Canadian exceptionalism, multiculturalism and acceptance:

*What is hard is that we throw things in our face based on what's happening in the states, …. and I'm not justifying any action that are happening in the States … However, as a Canadian police officer, why do I always have to answer to what they do down there? I'm not responsible for what's happening. Nor do we treat people, not the Canadian people treat the same way people here in Canada … What I love the most about Canada is because it's the most multicultural country and most accepting country, I think in the world. The [United] states, honestly, they're crazy down there.*

In this case, Officer Genevieve (White) underestimates systemic racism in Canadian policing by referring to race-neutral discourses about Canadian multiculturalism and belonging (Samuels-Wortley, 2021; Satzewich & Shaffir, 2009) to justify colorblind racism and deny police violence against racialized communities in Canada.

Officer Rachel (White) said she has seen officers say racist jokes on duty and yet, argues that police violence against people of color, especially Black people, in the United States is not a reflection of racism in Canada:

*I see what's happening in the states and how they're targeting black people, and people of color there. And I believe that's happening. I fully believe that it's happening. I feel like they have like such ingrained bias that they are scared when they encounter someone of a different race, because we've just been told that their whole life. I feel like the racism here [Canada] is different than that, I feel like it's just as socially unacceptable, joking or commentary kind of thing. […]I've definitely experienced racist banter and stuff like that if that makes sense and also biased commentary. So, you know, improper language, politically incorrect commentary, bias, but I have not experienced what I would perceive as a difference in service.*

Officer Rachel (White) uses the minimization of racism colorblind frame (Bonilla-Silva, 2022) to argue that systemic racism in Canadian policing is somehow less harmful than American policing because her colleagues usually say, *“racist like banter*” *“improper language*” and “*politically incorrect commentary*” whereas American police officers use violence on people of color. Officer Rachel (White) and Officer Genevieve's (White) argument about police violence in the United States not being a reflection of racism in Canada illustrates that Canadian police organizations label racialized police violence, especially anti-Black and anti-Indigenous police violence, as an American problem, reinforcing the myth that Canada is an inclusive and welcoming country that is not affected by racialized police violence (Etoroma, 2020; Glasbeek et al., 2020; Maynard, 2017; Samuels-Wortley, 2021). Canadian police agencies obscure the reality of systemic racism in policing by promoting racial diversity policies to present Canada as a place where racism is unheard of (Samuels-Wortley, 2021).

### Anti-Indigenous Racism

Women officers discussed anti-Indigenous racism in policing and racialized police violence against Indigenous people*.* Officer Charlotte (Black) worked in downtown and residing Inner-city neighborhoods for some time. While she was working in downtown, she witnessed a fair number of officers act disrespectful to unhoused Indigenous people:

*I think the biggest thing I’ve seen is working in downtown and seeing you know certain members that I've worked with just be completely disrespectful to homeless people. […] I think being the way some of those members treat some of its Indigenous communities, some of the homeless, many downtown was very frustrating and very disappointing. And yeah, I think just the way they treated them physically, emotionally, when they were dealing with some of them in different situations, but yeah, in a general basis, I would say that for sure.*

Officer Chloe (White, LGBTQIAA2S+) shared a similar narrative. Officer Chloe (White, LGBTQIAA2S+) worked in predominantly Indigenous neighborhoods at the beginning of her career. During that time, members of her squad held a lot of racist and derogatory perceptions about unhoused Indigenous peoples living in encampments. Officers often labeled unhoused Indigenous people as the “*scum of society who are kind of helpless*” and would pepper spray and tear down their tents:

*And I'm gonna say I've mostly experienced it with Indigenous populations, especially at the start of my career. And just being like, my perception and what I first thought was like a lot of Indigenous people, there was a lot that time I was there, there was some support resources in the area. So we'd get a lot of homeless, we get a lot of drugs, we get a lot of drinking, a lot of drug use a lot of camps and I'd say the mentality of the squad and the crew that I was on was that they were just kind of like that feeling of like the scum of society who are just kind of helpless, and let's go use pepper spray on their tents and rip down their tents, and not focus on getting them the resources and the help.*

This incident illustrates how officers emphasize warrior police culture (Sierra-Arevalo, 2019, 2021; Simon, 2023; Stoughton, 2014) to physically assault unhoused Indigenous people. The problematic racist assumptions police officers who worked with Officer Chloe (White, LGBTQIAA2S+) had about unhoused Indigenous peoples living in encampments contributes to the racialized policing of Indigenous peoples in public spaces (Comack, 2012; Freistadt, 2014, 2016; Laming, 2023; Palmater, 2016; Rudin, 2006). Police organizations and by-laws maintain racial hierarchies in urban, suburban, and rural communities by overpolicing and displacing unhoused Indigenous peoples into vulnerable, socioeconomically disadvantaged spaces (Freistadt, 2014). As a result, unhoused Indigenous peoples get targeted by the police and subjected to physical violence, anti-Indigenous racism, and harassment (Kauppi & Pallard, 2016).

Officer Amelia (Black) recalled a time when she met a 40-year-old Indigenous man who is a residential school survivor. When the Indigenous man started talking about his experiences of residential schooling, some officers immediately denied his experiences, claiming he was “*too young*” to be in the residential school system:

*…. Some guy was like, “when I was residential schooling” and whatnot. And then some people were just like, oh as if, he's only like, 40 something years old. And he was just, like, be you know, the last residential schooling closed in 96. You can just, you know, dismiss people. I don't think it's a reason for us to you know, to discredit anyone's experience or struggles that they went through because it just makes them feel shitty*.

Not only did Officer Amelia's (Black) colleagues deny the Indigenous man's experiences of residential schooling, but they also fail to acknowledge that the residential school system operated from the 1880s until 1996. The system forcibly removed Indigenous children from their families and prohibited them from practicing their Indigenous language and culture (Hanson et al., 2020). Children were subjected to physical and sexual violence for speaking their Indigenous language (Hanson et al., 2020). Residential school survivors remember “being beaten and strapped; some students were shackled to their beds, some had needles shoved in their tongue for speaking their native languages” (Hanson et al., 2020, p. 3). Canadian police organizations have a tendency of denying the role they played in establishing the residential school system (Chung, 2011). The Royal Canadian Mounted Police (RCMP) forcibly removed Indigenous children from their home and threatened to arrest Indigenous parents who refuse to send their children to residential schools (LeBeuf, 2011). The RCMP helped Indian Agents and churches in searching and returning Indigenous children who attempted to escape and when there was evidence of physical and sexual abuse in the residential school system, the RCMP never investigated the incidents (LeBeuf, 2011). Canadian police organizations played an important role in protecting the interests of the white settler colonial state (Comack, 2012; Flores & Alfaro, 2023; Laming, 2023; Monchalin, 2016; Nettlebeck & Smandych, 2010).

When I asked how police culture affects the way officers interact with members of the public, Officer Sarah (White) talked about a time when one of her trainers racially profiled five Indigenous people in a vehicle: “*I was with one of my trainers and he saw a car of five Indigenous people in the car, and he said, ‘we should pull them over.’ I said, why? And he's like because there's five Indigenous people in the car. So again, that's not a reason to pull someone over.*”

One of the passengers ended up being charged with impaired driving, but Officer Sarah (White) argues that her trainer still had no right to pull over a passenger because they’re Indigenous: “*In conclusion, it actually turned out that one of them was drinking and driving and you know, he got an impair charge. Did the end justify the means? I don't know. Technically, like, that's not a reason to pull them over just because they're Indigenous.*”

The example Officer Sarah (White) provides about the suspicion her trainer had about Indigenous passengers in the vehicle illustrates that police culture is embedded in racialized policing practices such as stop and search and use of force (Epp et al., 2014; Owusu-Bempah & Gabbidon, 2021). Police culture socializes officers such as Sarah's trainer to be suspicious (Skolnick, 1966), about the Indigenous community, an approach that leads them to develop racist stereotypes about Indigenous peoples behaviors and appearances as indicators of committing crime (Flores & Alfaro, 2023).

### Anti-Black Racism

Women officers discussed anti-Black racism in Canadian policing and the racial tensions between the police and Black communities. Officer Chloe (White, LGBTQIAA2S+) said the Black community and the local Black Lives Matter organization don’t have a good relationship with the police: “*It's just there's been I feel this double-sided pushback from the membership against the communities just feeling like all these communities. These underrepresented groups are the black community they are Black Lives Matter group they hate police ….*”

Officer Amelia (Black) talked about how anti-Black racism in policing negatively affects the Somali community. She said the Somali community doesn’t have a good relationship with the police, especially when it comes to addressing the prevalence of gun violence among young Somali men and boys: “*I don't know how much you know about the Somali community and the police force here; they don't have great relations. And in our community, unfortunately, we have a lot of gun violence. And it's the young youth males. And so, there's a lot of stress between the police as well as the community.*”

Despite the prevalence of anti-Black racism in Canadian policing, some White women officers denied anti-Black racism in Canadian policing. One White women officer denied anti-Black racism in Canadian policing by utilizing racialized cultural schemas about Blackness, the Somali community, and crime. Officer Brooklyn (White) claims that families of homicide victims, including those from the Somali community don’t cooperate with the police because of their criminal behavior. Officer Brooklyn (White) goes as far as using intersectionality to justify colorblind racism and anti-Blackness in policing, arguing that race and ethnicity is not a factor when it comes to homicide investigation, but rather the criminal lifestyles of Somali Canadians. She further states that the witnesses of the victims don’t cooperative with the police, regardless of race and ethnicity:

*So, it's not that the Somali community is being uncooperative a lot of people that are involved in criminal lifestyle. Right, so the actual intersection is not the race the intersectionality there is the criminal lifestyle. So, there's a lot of people in a criminal lifestyle that end up getting murdered because of their lifestyle, not because they're Somali or east African right there being murdered because they're involved in, and their families are as equally uncooperative with us. And the witnesses are equally as uncooperative with us as a lot of other ethnicities.*

Officer Brooklyn (White) uses the cultural racism colorblind frame (Bonilla-Silva, 2022) to argue that Somali Canadians have a “*criminal lifestyle.*” The perception that Somali and other East Africans are involved in “*criminal lifestyles*” asserts a culturally racist belief that certain Black communities, such as the Somali community, are more prone to getting involved in criminal activities than others (Maynard, 2017). The culturally racist beliefs Officer Brooklyn (White) has about Black people, particularly Somali Canadians parallels with previous studies that prove police officers use racist stereotypes about Black people when investigating Black homicide victims (Macpherson, 1999). Police culture reinforces racist stereotypes about Black people, associating Blackness with criminality (Duhaney, 2022a; Gordon, 2024; Macpherson, 1999; Maynard, 2017).

Other White women officers denied anti-Black racism in Canadian policing by erasing the role race plays in investigating Black homicide victims. Officer Mackenzie (White), like Officer Brooklyn (White) also denied anti-Black racism in homicide investigation, claiming that the public don’t understand systemic racism in Canadian policing. When she worked as a homicide detective, she argues that race was never a factor in investigating the murders of Black and Indigenous people:

*I think there's definitely a misunderstanding from the public of what systemic racism is. And it's taking a real mental toll on our membership. Because having been in homicide where I've investigated murders of Black women, Black men, Indigenous males, females … At no point was it [race] ever a factor, and a lot of families have no idea that I've been in, I've been involved in investigation for 18 h, and I'm exhausted. And then I see a picture of the person and I had no idea that they were Black, or I had no idea they're Indigenous, because I'm just doing my job, right?*

Not only do Officer Mackenize (White) and Officer Brooklyn (White) deny racism in homicide investigations, but they also completely overlook the structural factors of homicide in Black and Indigenous communities. Indigenous scholars argue that colonialism, racism, and patriarchy are structural factors that lead to homicide in Indigenous communities and missing and murdered Indigenous women, girls and LGBTQIAA2S+ people in Canada and the United States (Flores & Alfaro, 2023; Monchalin et al., 2019). Black Canadians comprise only 4.3% of the Canadian population and yet are 4 times more likely to be victims of homicide compared to White people (Statistics Canada, 2024). White women officers in the study were consistent in their beliefs about police work, arguing that policing is a “race-neutral practice” (Maynard, 2017, p. 86), and therefore believe that racism has no effect on police investigative practices (Foster, 2008; Welsh et al., 2021). White officers often deny racial disparities in policing practices (Gordon, 2024; Welsh et al., 2021), arguing that they “don’t see color, just people” (Bonilla-Silva, 2022, p. 1).

### Xenophobia and Racism

A few Black women officers reported that the police hold xenophobic and racist perceptions about refugees. Officer Amelia (Black) recalled a time when she was responding to a call from a racialized community with many refugees. One of her colleagues started shouting racist and xenophobic slurs to a group of refugees, blaming Prime Minister Justin Trudeau and the Canadian government for allowing refugees into the country:

*So, like, I’ve definitely experienced some things that I've called them out of like, there's one time we were dealing with an incident. It was from a certain community, and a member tried to blame Trudeau for allowing that community into the [country], refugees, blaming refugees. I looked at him, excuse me, I was like, that's not why he did what he did is because he's from a certain community, that's not how that works. And unfortunately, he just made it, you know what I mean? clearly, it's him as an individual, not for his whole community, that's why part of it made no sense. How many people do I deal with? If I dealt with a white person from a white community, do I now blame the whole white community for their actions?*

The officer culturally framed all refugees as criminals and blamed the entire community for the individual's criminal offense. Officer Amelia (Black) replied to the officer's racist and xenophobic comment, arguing that similar accusations wouldn’t have been said about a White person from a predominantly white community. The example Officer Amelia (Black) provides about racism and xenophobia toward refugees demonstrates that officers use cultural racism (Bonilla-Silva, 2022) to reinforce the culturally racist idea that crime is a result of race, ethnicity, and migration (Bowling et al., 2001).

## Discussion and Conclusion

This study offers a comprehensive analysis on how police culture reinforces systemic racism in Canadian policing. The findings contribute to the literature on colorblind racism, intersectionality, and police culture. These findings reveal that whiteness is not a monolith (Carbado, 2019; Ferber, 2007; Frankenberg, 1993) and therefore, White women officers also contribute to systemic racism in policing. Most White women officers in the study, expect for a few officers, used colorblind racist frames (Bonilla-Silva, 2022) to minimize or deny the role police culture plays in shaping systemic racism in Canadian policing. For instance, two White women officers used the minimization of racism frame (Bonilla-Silva, 2022) to argue that systemic racism in Canadian policing is somehow less harmful than American policing because of Canadian multiculturalism and racist jokes, a common police cultural practice (Pérez, 2022; Pérez & Ward, 2019; Waddington, 1999). One White women officer used the cultural racism frame (Bonilla-Silva, 2022) to justify anti-Black racism in Canadian policing, arguing that certain Black communities in Canada such as the Somali community are victims of homicide because they have a “*criminal lifestyle*.” White women officers consistently argued that policing is race-neutral, denying the role racism plays in policing practices such as stop and search (Epp et al., 2014). In contrast, Black and Biracial (Indigenous/White) women officers generally acknowledged how police culture reinforces systemic racism in Canadian policing.

Findings from this research advance more discussions about systemic racism in Canadian policing. Black, Indigenous and other racialized communities in Canada have for many years expressed concerns about systemic racism in Canadian policing and its racialized policing practices, such as carding and use of force (Stelkia, 2020; Wortley & Owusu-Bempah, 2022). These findings illustrate how Canadian police organizations overpolice Indigenous communities, often resulting in racialized police violence, criminalization, injury or even death and when Indigenous people call the police for assistance, they receive little to no protection (Flores & Alfaro, 2023; Laming, 2023; Palmater, 2016; Rudin, 2006). For example, women officers described how officers emphasize warrior police culture and suspiciousness to physically abuse unhoused Indigenous people and racially profile Indigenous people during traffic stops. Like the police killings of Black Americans, Black Canadians also experience anti-Black racism in Canadian policing, especially in racial profiling and police shootings (Maynard, 2017; Tator & Henry, 2006; Wortley & Owusu-Bempah, 2022). The findings illustrate that police culture reinforces officers to associate Blackness with criminality by utilizing racialized cultural frameworks to justify anti-Black racism in Canadian policing. Despite Canada portraying themselves as a nation that values multiculturalism and belonging (Samuels-Wortley, 2021), the findings shows that officers use cultural racism (Bonilla-Silva, 2022) and xenophobia to frame refugees as criminals.

There are a few limitations to the study that are important to note. A limitation of the study is the small sample size (16 women officers) and lack of racial diversity among the participants. The skewed racial demographics of the study may have influenced what was shared with me: 75 percent (12 out of 16) of participants in the study were White women, 18% (3 out of 16) were Black women and 6% (1 of 16) were Biracial (Indigenous/White) women. LGBTQ2SIA+ women officers represented 25% (four out of 16) of the sample, and they all racially identify as White. The study focused only on one police organization. Recruiting participants was done with the help of the police research unit. Recruiting women officers through internal police channels may introduce social desirability bias from officers who volunteered to participate in the research study and the type of officers who were likely to participate in the study than others. This recruitment approach may have influenced the responses given by the participants in the study. These limitations notwithstanding, my findings shed light on how police culture perpetuates systemic racism in Canadian policing and makes an important contribution to the Canadian literature on race and policing.

Future research should look further into examining how police culture reinforces systemic racism in Canadian policing by conducting a larger set of interviews with police officers from one Canadian city or across the country. Ethnographic research would be useful to uncover more about how police culture shapes systemic racism in Canadian policing. Researchers can conduct several months of observations in police ride-alongs, preshift meetings and street patrol. This approach can help researchers take fieldnotes of how police officers encounter Black, Indigenous, and other racialized communities. Alternatively, surveys can provide valuable quantitative data on police culture and racism in Canadian police services. Conducting interviews with people from Black, Indigenous, and other racialized communities is another critical way of understanding the culture of policing and systemic racism in Canadian policing.
